# Cell plasticity in wound healing: paracrine factors of M1/ M2 polarized macrophages influence the phenotypical state of dermal fibroblasts

**DOI:** 10.1186/1478-811X-11-29

**Published:** 2013-04-19

**Authors:** Diana TA Ploeger, Nynke A Hosper, Martin Schipper, Jasper A Koerts, Saskia de Rond, Ruud A Bank

**Affiliations:** 1 Department of Pathology and Medical Biology, Medical Biology Section, University Medical Center Groningen, University of Groningen, Hanzeplein 1, Groningen 9713 GZ, The Netherlands

**Keywords:** Cell plasticity, Classically/alternatively activated macrophages, Extracellular matrix remodeling, Inflammation, Matrix metalloproteinases, Paracrine signaling, Primary human dermal fibroblasts

## Abstract

**Background:**

Macrophages and fibroblasts are two major players in tissue repair and fibrosis. Despite the relevance of macrophages and fibroblasts in tissue homeostasis, remarkably little is known whether macrophages are able to influence the properties of fibroblasts. Here we investigated the role of paracrine factors secreted by classically activated (M1) and alternatively activated (M2) human macrophages on human dermal fibroblasts (HDFs).

**Results:**

HDFs stimulated with paracrine factors from M1 macrophages showed a 10 to > 100-fold increase in the expression of the inflammatory cytokines IL6, CCL2 and CCL7 and the matrix metalloproteinases MMP1 and MMP3. This indicates that factors produced by M1 macrophages induce a fibroblast phenotype with pro-inflammatory and extracellular matrix (ECM) degrading properties. HDFs stimulated with paracrine factors secreted by M2 macrophages displayed an increased proliferation rate. Interestingly, the M1-activated pro-inflammatory fibroblasts downregulated, after exposure to paracrine factors produced by M2 macrophages or non-conditioned media, the inflammatory markers as well as MMPs and upregulated their collagen production.

**Conclusions:**

Paracrine factors of M1 or M2 polarized macrophages induced different phenotypes of HDFs and the HDF phenotypes can in turn be reversed, pointing to a high dynamic plasticity of fibroblasts in the different phases of tissue repair.

## Background

In wound healing and fibrosis, a variety of processes are crucial, such as inflammation, cell proliferation, cell migration and extracellular matrix (ECM) remodeling. Two major cellular players in these processes are macrophages and fibroblasts [1-4]. During the proliferation phase of wound healing, fibroblasts proliferate and migrate into the wound site to form granulation tissue. Part of these fibroblasts differentiate into myofibroblasts and produce new ECM, mainly in the form of collagen, which is necessary to support cellular ingrowth. The degradation of collagen in the wound is mainly controlled by matrix metalloproteinases (MMPs). In normal wound healing, most of the myofibroblasts and fibroblasts go into apoptosis in due time, or leave the wound site. However, in fibrosis myofibroblasts accumulate and produce an excess of collagen that remains deposited, thereby causing damage to the tissue architecture and diminishing its function [5-9].

The other important cell type in wound healing and fibrosis, macrophages, exist as resident tissue-specific macrophages, or are derived from circulating blood monocytes that undergo diapedesis and subsequently differentiate into macrophages. Macrophages display various activation states. The two opposite activation states are known as classically activated (M1) and alternatively activated (M2) macrophages [10,11]. The M1 macrophage is pro-inflammatory and is often associated with tissue injury and inflammation, whereas the M2 macrophage is associated with tissue repair and fibrosis [12-15]. Factors that induce the M1 polarization of macrophages are interferon gamma (INFG), tumor necrosis factor (TNF), and/or lipopolysaccharides (LPS), whereas M2 macrophage polarization is induced by interleukin 4 (IL4), 13 (IL13), 10 (IL10), glucocorticoids and/or transforming growth factor beta 1 (TGFB1) [11,16,17].

In the inflammatory phase of wound healing, invading macrophages are pro-inflammatory (M1) and secrete several cytokines and chemokines, like chemokine (C-C motif) ligand 2 (CCL2) (monocyte chemotactic protein-1), CCL7 (monocyte chemotactic protein-3) and interleukin 6 (IL6). These cytokines/chemokines play a crucial role in wound healing and are involved in fibrogenesis [13,15,18-21]. M2 macrophages are associated with the healing process by modulating the inflammatory process and by secreting factors like CCL18. CCL18 is able to stimulate fibroblast proliferation and collagen production, which are important in the healing process, but an increased CCL18 expression can also induce fibrosis [22,23].

It has been shown that macrophages show a high dynamic plasticity. Macrophages can change, depending on the stimulus in the micro-environment, their secretion pattern of cytokines and chemokines several times [24-26]. For example, human primary M1 polarized macrophages can be re-polarized by secreted factors from their own counterparts, M2 macrophages, and *vice versa*, *in vitro*[27]. *In vivo*, there are indications that re-polarization of macrophages also occurs, as shown in a mouse model for atherosclerosis [28] and in a rodent model for myocardial infarction [29]. This macrophage plasticity not only has an effect on the inflammation phase of wound healing, but likely also on the proliferation and remodeling phase.

Despite the relevance of macrophages and fibroblasts in tissue homeostasis, remarkably little is known whether the different types of human primary macrophages are able to influence directly the properties of human primary fibroblasts. Most of the data found in literature have generally been generated with cell lines [30-32] or primary cells from murine origin [33,34], mostly without paying attention to the M1/M2 activation state. Here we investigated the role of paracrine factors secreted by human M1 and M2 macrophages on primary adult human dermal fibroblasts (HDFs) with respect to proliferation, myofibroblast formation, collagen synthesis and degradation, as well as synthesis of various cytokines. Because of the plasticity of macrophages, we also set out to investigate the influence of paracrine factors secreted by M1 macrophages followed by paracrine factors secreted by M2 macrophages on HDFs.

## Results

### Characterization of macrophages after M1 or M2 polarization

**Figure 1:**
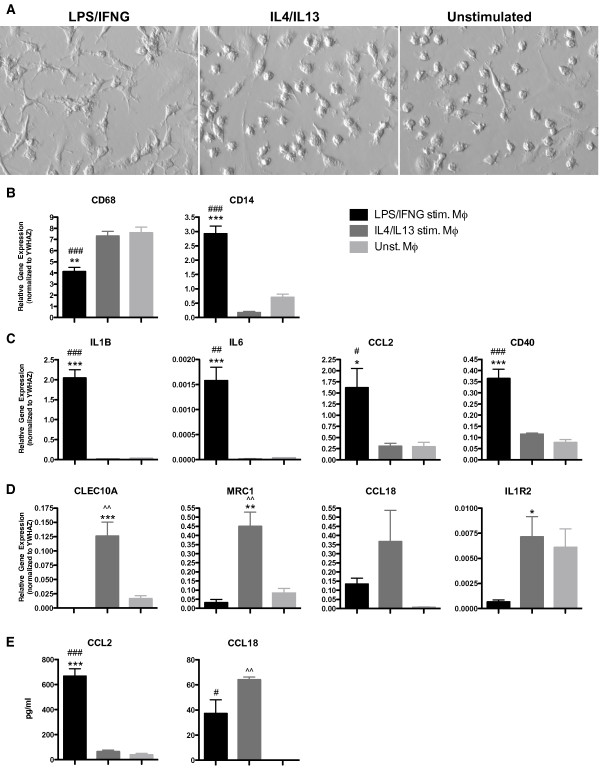
**Characterization of macrophages after M1 or M2 polarization.** After stimulation with LPS/IFNG, M1 macrophages showed a dendritic morphology while IL4/IL13 (M2) stimulated and unstimulated macrophages showed a rounded and/or spindle-shaped morphology (**A**). The three primary macrophages subsets showed, compared to reference gene YWHAZ, a high expression of CD68. In M1 polarized macrophages the CD68 gene expression is downregulated while the expression of CD14 is upregulated compared to M2 or unstimulated macrophages (**B**). LPS/IFNG-stimulated (M1) macrophages showed upregulated gene expression of IL1B, IL6, CCL2 and CD40 (**C**). IL4/IL13-stimulated (M2) macrophages upregulated the gene expression of CLEC10A, MRC1 and tended to upregulate CCL18. IL1R2 showed a high expression in M2 and unstimulated macrophages and was downregulated in M1 polarized macrophages (**D**). At protein level, more CCL2 was observed in conditioned medium from M1 macrophages. CCL18 protein secretion showed, like CCL2, values that correlated with gene expression (**E**). * p < 0.05, Difference between LPS/IFNG and IL4/IL13 stimulated macrophages, ** p < 0.01, *** p < 0.001. # p < 0.05, Difference between LPS/IFNG stimulated and unstimulated macrophages, ### p < 0.001. ^^ p < 0.01, Difference between IL4/IL13 stimulated and unstimulated macrophages. Data were analyzed using one-way ANOVA followed by Tukey’s post-test. Gene expression analysis n = 4, protein secretion n = 3.

Primary human macrophages responded to LPS/IFNG or IL4/IL13, resulting in M1 or M2 polarization, respectively. M1 polarized macrophages adopted a “dendritic”-like morphology with large filopodia while M2 polarized macrophages showed a rounded and/or spindle-shaped morphology, which was comparable with the morphology of unstimulated macrophages (Figure 1A).

The three macrophage subsets showed compared to the reference gene tyrosine 3-monooxygenase/tryptophan 5-monooxygenase activation protein, zeta polypeptide (YWHAZ), a high expression of CD68, which is a general marker for macrophages. M1 macrophages had a lower CD68 expression than M2 polarized or unstimulated macrophages (Figure 1B). CD14, a co-receptor for toll-like receptor 4 (TLR4), is involved in LPS recognition and is upregulated by M1 polarized macrophages compared to M2 or unstimulated macrophages (Figure 1B).

Macrophages stimulated for 48 h with LPS/IFNG showed an upregulation of the inflammatory genes interleukin 1 beta (IL1B), IL6 and CCL2 compared to M2 polarized and unstimulated macrophages (Figure 1C). A similar upregulation of CD40, a protein involved in the activation of antigen presenting cells, was seen after LPS/IFNG stimulation (Figure 1C).

Macrophages stimulated with IL4/IL13 showed an upregulated gene expression of C-type lectin domain family 10, member A (CLEC10A; also known as macrophage galactose N-acetyl-galactosamine specific lectin) and mannose receptor, C type 1 (MRC1) compared to M1 polarized or unstimulated macrophages. CCL18 tended to be upregulated in IL4/IL13 stimulated macrophages while interleukin 1 receptor, type II (IL1R2), which acts as a decoy receptor for the type I interleukin 1, showed a higher expression in IL4/IL13 and unstimulated macrophages than M1 polarized macrophages (Figure 1D). M1 macrophages secreted significantly more CCL2 compared to M2 and unstimulated macrophages. M2 and M1 macrophages secreted more CCL18 compared to unstimulated macrophages, but no significant differences in secretion were seen between M1 and M2 (Figure 1E). M1 macrophages secreted more pro-inflammatory cytokines and chemokines compared to M2 and unstimulated macrophages (Table 1). M2 macrophages secreted fibroblast growth factor 2 (FGF2), which was significant different compared to M1 and unstimulated macrophages (Table 1).

**Table 1:** Overview secreted cytokines, chemokines and growth factors by different polarized macrophages

	**Secreted factors by macrophages (pg/ml)**			***Ratio***		
**Protein symbol**	**M1**	**M2**	**Unst.**	**M1:M2**	**M1:Unst.**	**M2:Unst**
CCL2	743 ± 123	0	0	743	743	1
CCL3	45 ± 28	0	0	45	45	1
CCL4	192 ± 59	0	0	192	192	1
CCL5	26 ± 9	0	0	26	26	1
CCL18	37 ±19	64 ± 4	0	−2	37	64
CXCL9	183 ± 80	0	0	183	183	1
CXCL10	370 ± 83	7,4 ± 1,9	7,4 ± 1,2	50	50	1
FGF2	1,6 ± 3,2	10 ± 4	0	−6	1	10
IL6	31 ± 7	0	0	31	31	1
IL8	1171 ± 388	52 ± 11	63 ± 19	23	19	1
IL12p40/p70	84 ± 30	0	0	84	84	1
IL15	40 ± 11	0	0	40	40	1

Overall, our results indicate that M1 polarized macrophages were pro-inflammatory while M2 polarized macrophages were non-inflammatory and unstimulated macrophages adopted a M2 “intermediate” phenotype.

### Morphology of HDFs stimulated with conditioned medium (CM) of M1 polarized, M2 polarized, or unstimulated macrophages

**Figure 2:**
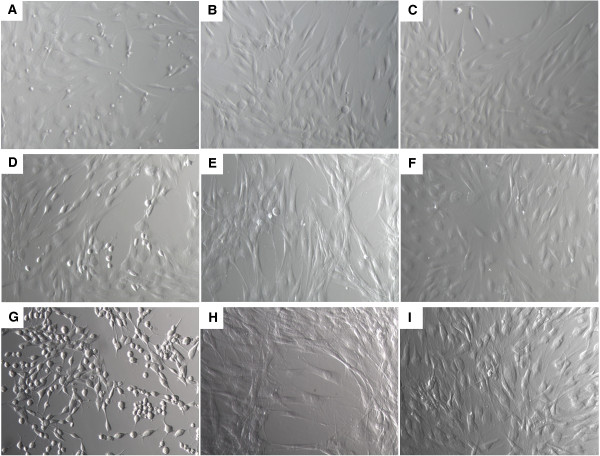
**Morphology of HDFs stimulated with CM of M1 polarized, M2 polarized, or unstimulated macrophages.** HDFs stimulated with CM of M1 polarized macrophages; 24 h (**A**), 72 h (**D**), and 144 h (**G**). After 24 h, most of the fibroblasts showed a spindle-shaped morphology although some rounded fibroblasts were seen. After 72 h, the fibroblasts adopted a rounded morphology that was most prominent after 144 h. HDFs stimulated with CM of M2 polarized macrophages; 24 h (**B**), 72 h (**E**) and 144 h (**H**). After 24 h the cells showed a spindle-like morphology, which was changed into an elongated spindle-like morphology after 72 h. HDFs stimulated with CM from unstimulated macrophages 24 h (**C**), 72 h (**F**), and 144 h (**I**). The fibroblasts showed a spindle-like morphology after 24 h, which was not changed in time.

Dermal fibroblasts were stimulated with CM of M1 polarized, M2 polarized or unstimulated macrophages for 24 h, 48 h, 72 h and 144 h. After 24 h of stimulation, the fibroblasts showed a spindle-shaped morphology in all three conditions (Figure 2A, B, C). After 24 h of stimulation with CM of M1 macrophages some rounded fibroblasts were seen, which were not present in the fibroblast cultures stimulated with CM of M2 polarized or unstimulated macrophages (Figure 2A). After 48 h of stimulation, the morphology of the fibroblasts was similar to that of 24 h of stimulation (data not shown). However, the fibroblast morphology changes in time. CM of M1 macrophages induced a rounded morphology, which was clearly seen after 72 h (Figure 2D) and 144 h (Figure 2G), while fibroblasts stimulated with CM of M2 macrophages adopted an elongated spindle-shaped cell morphology after 72 h and 144 h (Figure 2E, and H). The morphology of fibroblasts stimulated with CM of unstimulated macrophages had a spindle-shaped morphology after 72 h and 144 h (Figure 2F and I) that was similar to 24 h (Figure 2C). This morphology was also seen by fibroblasts cultured in control medium (data not shown).

### CM from M1 macrophages induces a pro-inflammatory HDF

**Figure 3:**
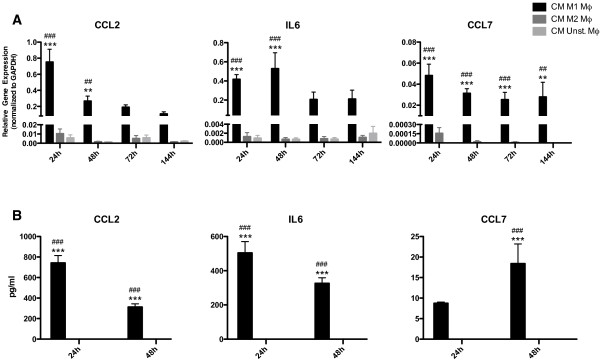
**CM from M1 macrophages induces a pro-inflammatory HDF.** HDFs upregulated the gene expression of pro-inflammatory genes CCL2, IL6 and CCL7 after stimulation with CM of M1 polarized macrophages compared to M2 polarized and unstimulated macrophages (**A**). HDFs stimulated with CM of M1 macrophages secreted significantly more CCL2, IL6 and CCL7 after 24 h and 48 h, whereas secretion levels of these proteins by fibroblasts stimulated with CM of M2 macrophages or unstimulated macrophages were below the detection limit (**B**). ** p < 0.01, Difference between HDFs stimulated with CM of M1 polarized and CM of M2 polarized macrophages, *** p < 0.001. # p < 0.05, Difference between HDFs stimulated with CM of M1 polarized and CM of unstimulated macrophages, ## p < 0.01, ### p < 0.001. Gene expression analysis data were analyzed using two-way ANOVA followed by Bonferroni’s post-test, n = 4. Protein secretion data were analyzed using one-way ANOVA followed by Tukey’s post-test n = 3.

HDFs showed, after stimulation with CM of M1 macrophages, a > 10-fold increase in the expression of the pro-inflammatory gene CCL2 compared to fibroblasts stimulated with CM of M2 or unstimulated macrophages at all time points (Figure 3A). The expression of the pro-inflammatory genes IL6 and CCL7 was > 100-fold upregulated at all time points by fibroblasts stimulated with CM of M1 macrophages compared to fibroblasts stimulated with CM of M2 or unstimulated macrophages (Figure 3A).

Secretion of the cytokines CCL2, IL6 and chemokine CCL7 by dermal fibroblasts was determined after 24 h and 48h of stimulation. Fibroblasts stimulated with CM of M1 macrophages secreted significantly more CCL2 and IL6 compared to fibroblasts stimulated with CM of M2 macrophages or unstimulated macrophages after 24 h and 48 h (Figure 3B). Secretion of CCL7 by M1 CM stimulated fibroblasts was higher after 24 h and becomes significant after 48 h of stimulation compared to fibroblasts stimulated with M2 or unstimulated macrophages CM (Figure 3B). These results are in accordance with the gene expression patterns of the stimulated fibroblasts.

The results indicate that M1 macrophages induce, by means of paracrine signaling, a pro-inflammatory dermal fibroblast.

### CM from M1 macrophages induces the expression of ECM degrading enzymes by HDFs

**Figure 4:**
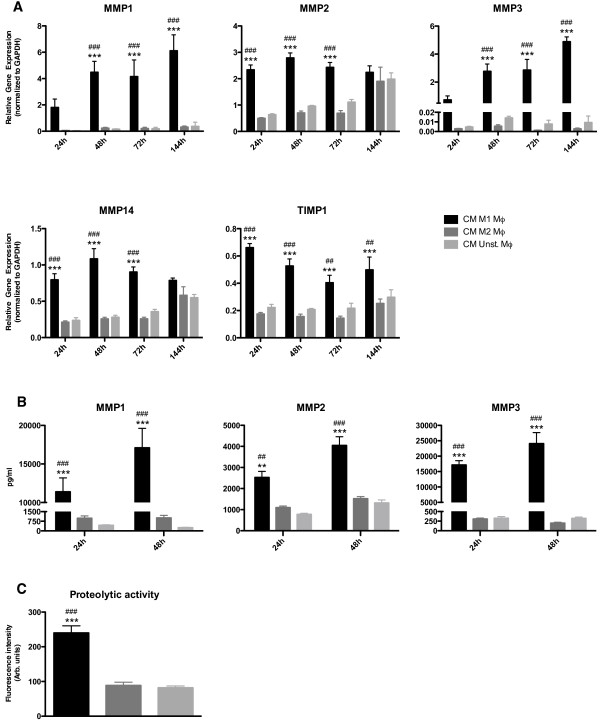
**CM from M1 macrophages induces dermal fibroblasts with extracellular matrix degradation properties.** HDFs upregulated the gene expression of MMP1, MMP2, MMP3, MMP14 and TIMP1 after stimulation with CM of M1 polarized macrophages compared to M2 polarized and unstimulated macrophages (**A**). HDFs stimulated with CM of M1 macrophages secreted significantly more MMP1, MMP2 and MMP3 protein compared to fibroblasts stimulated with CM of M2 macrophages or unstimulated macrophages (**B**). HDFs stimulated with M1 CM showed a higher net proteolytic activity compared to HDFs stimulated with CM of M2 and unstimulated macrophages (**C**). ** p < 0.01, Difference between HDFs stimulated with CM of M1 polarized and CM of M2 polarized macrophages, *** p < 0.001. ## p < 0.01, Difference between HDFs stimulated with CM of M1 polarized and CM of unstimulated macrophages, ### p < 0.001. Gene expression analysis data were analyzed using two-way ANOVA followed by Bonferroni’s post-test, n = 4. MMP secretion was analyzed using one-way ANOVA followed by Tukey’s post-test, n = 3. Proteolytic activity was analyzed using one-way ANOVA followed by Tukey’s post-test, n = 4.

Stimulation of dermal fibroblasts with CM of M1 macrophages already showed an upregulated gene expression of MMP1, MMP2, MMP3 and MMP14 compared to the other conditions after 24 h (Figure 4A). These MMP gene expression profiles were consistently upregulated over time, except for MMP2 and MMP14 after 144 h. Tissue inhibitor of metalloproteinases −1 (TIMP1) was also upregulated (2–3 fold) in fibroblasts stimulated with CM of M1 macrophages, but the total MMP gene expression levels were much higher upregulated: MMP1 and MMP3 were > 10 and > 100 fold upregulated, respectively (Figure 4A). On protein level, the secretion of MMP1, MMP2 and MMP3 were upregulated by fibroblasts after stimulation with CM of M1 macrophages in the same order of magnitude as observed by the respective expression data (Figure 4B). Indeed, the secreted MMPs showed a higher net proteolytic activity compared to medium derived from fibroblasts stimulated with CM of M2 or unstimulated macrophages (Figure 4C).

The results indicate that fibroblasts subjected to factors produced by M1 macrophages show enhanced ECM degradation properties.

### CM of M1 polarized, M2 polarized or unstimulated macrophages does not induce myofibroblast differentiation of HDFs

**Figure 5:**
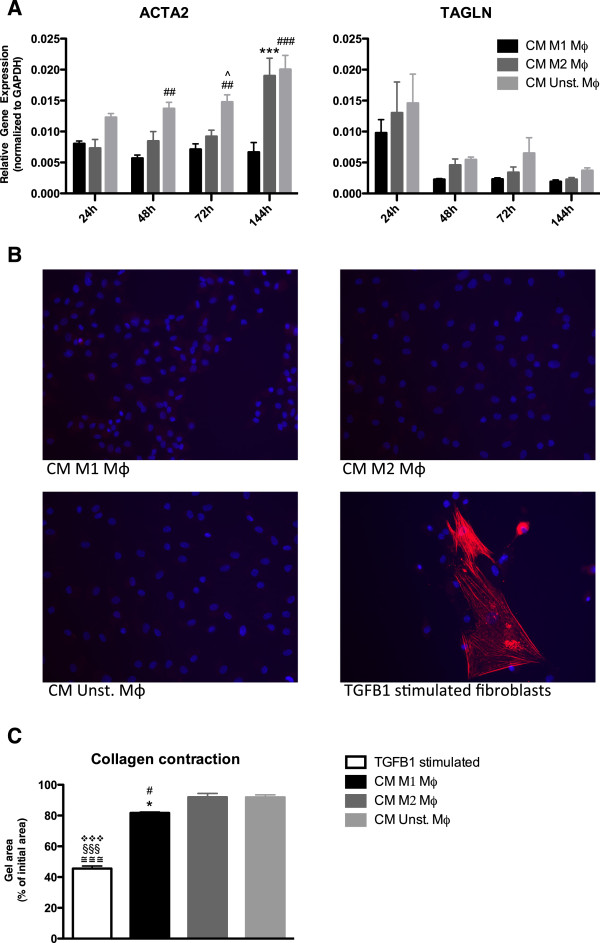
**CM of M1 polarized, M2 polarized or unstimulated macrophages do not induce myofibroblast differentiation of HDFs.** HDFs stimulated with CM of unstimulated macrophages showed, compared to fibroblasts stimulated with CM of M1 polarized macrophages, an upregulated gene expression of ACTA2 after 48 h, 72 h and 144 h. HDFs stimulated with CM of M2 polarized macrophages showed a higher gene expression of ACTA2 compared to HDFs stimulated with CM of M1 macrophages after 144 h (**A**). No differences were observed in TAGLN gene expression between the three conditions in all time points (**A**). No differences in ACTA2 protein expression were seen between the three conditions after 144 h. TGFB1 stimulated fibroblasts were used as positive control (**B**). Fibroblasts stimulated with CM of M1 macrophages contract the collagen 10% more than fibroblasts stimulated with CM of M2 and unstimulated fibroblasts. Fibroblasts stimulated with TGFB1 contract the collagen 50% more compared to the other stimulations (**C**). * p < 0.05, Difference between HDFs stimulated with CM of M1 polarized and CM of M2 polarized macrophages, *** p < 0.001. # p < 0.05, Difference between HDFs stimulated with CM of M1 polarized and CM of unstimulated macrophages, ## p < 0.01, ### p < 0.001. ^ p < 0.05, Difference between HDFs stimulated with CM of M2 polarized and CM of unstimulated macrophages. ≅≅≅ p < 0.001, Difference between HDFs stimulated with TGFB1 and CM of M1 polarized macrophages. §§§ p < 0.001, Difference between HDFs stimulated with TGFB1 and CM of M2 polarized macrophages. ❖❖❖ p < 0.001, Difference between HDFs stimulated with TGFB1 and CM of unstimulated polarized macrophages. Gene expression analysis data were analyzed using two-way ANOVA followed by Bonferroni’s post-test, n = 4. ACTA2 protein expression was shown in red and nuclei in blue (DAPI), original magnification 200×. Collagen gel contraction analysis data were analyzed using one-way ANOVA followed by Tukey’s post-test, n = 3.

Alpha-actin-2 (ACTA2; also known as alpha Smooth Muscle Actin), a marker for myofibroblast formation, is upregulated at gene expression level by fibroblasts stimulated with CM of unstimulated macrophages compared to CM of M1 stimulated macrophages after 48 h, 72 h and 144 h. Fibroblasts stimulated with CM of M2 macrophages showed an upregulation of ACTA2 compared to fibroblasts stimulated with CM of M1 macrophages after 144 h (Figure 5A). No differences were observed in transgelin (TAGLN) (smooth muscle protein 22-alpha) gene expression, a calponin that is mainly expressed by smooth muscle cells and myofibroblasts (Figure 5A).

On protein level no ACTA2 was seen in fibroblasts after 144 h of stimulation with the three different CM. This was in contrast to TGFB1 stimulated fibroblasts (myofibroblasts), which showed ACTA2 protein expression after 144 h (Figure 5B). TGFB1 stimulated fibroblasts showed a higher contractile force compared with fibroblasts stimulated with CM of different macrophages in a collagen gel contraction assay (Figure 5C). Fibroblasts stimulated with CM of M1 macrophages contract the collagen gel slightly more than fibroblasts stimulated with CM of M2 and unstimulated fibroblasts. It is reported by Zhu et al. that active MMPs increases collagen gel contraction [35,36]. It is likely that the secretion of active MMPs by fibroblasts stimulated with M1 CM causes the observed gel contraction.

Together, these results indicate that CM from M1, M2 or unstimulated macrophages did not result in the differentiation of fibroblasts into myofibroblasts.

### Proliferation of HDFs is induced by CM of M2 macrophages

**Figure 6:**
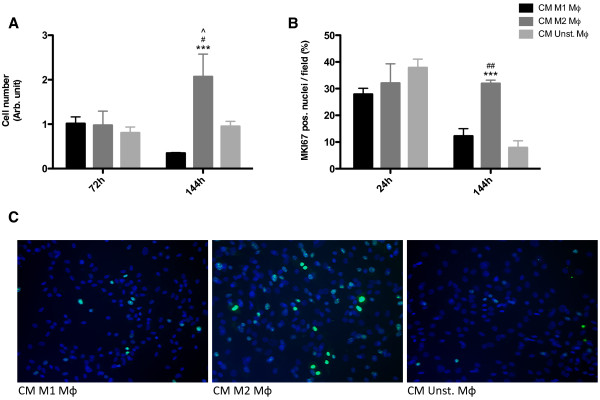
**Proliferation of HDFs is induced by CM of M2 macrophages.** After 72 h, no differences in cell numbers were seen between HDFs stimulated with CM of M1 polarized, M2 polarized or unstimulated macrophages. After 144 h, stimulation with CM of M2 macrophages increased the dermal fibroblast cell number, exclusively (**A**). This is due to proliferation of the HDFs as shown by MKI67 protein staining (**B**). More MKI67 positive nuclei (green) were seen in fibroblasts stimulated with CM of M2 macrophages compared to fibroblasts stimulated with CM of M1 and unstimulated macrophages after 144 h (**C**). *** p < 0.001, Difference between HDFs stimulated with CM of M1 polarized and CM of M2 polarized macrophages. # p < 0.05, Difference between HDFs stimulated with CM of M2 polarized and CM of unstimulated macrophages. ^ p < 0.05, Difference between 72 h and 144 h after stimulation of HDFs with CM of M2 polarized macrophages. Cell numbers and MKI67 positive nuclei were analyzed using two-way ANOVA followed by Bonferroni’s post-test n = 3. MKI67 protein expression was shown in green, nuclei in blue (DAPI) and original magnification was 200×.

After 72 h, fibroblast cell numbers were similar in all conditions, but increased exclusively after stimulation with CM of M2 macrophages after 144 h (Figure 6A). Nuclear protein Ki-67 (MKI67), a cellular marker for proliferation, showed the same amount of positive nuclei at 24 h in all conditions. This indicates that a comparable proliferation rate occurs at 24 h (Figure 6B). At 144 h, more MKI67 positive nuclei were seen when fibroblasts were stimulated with CM of M2 macrophages compared to CM from M1 or unstimulated macrophages, although in all three conditions positive nuclei were seen (Figure 6B and C).

The results indicate that CM from M2 macrophages induced proliferation of fibroblasts.

### Influence of CM of M1 polarized, M2 polarized or unstimulated macrophages on extracellular matrix deposition by HDFs

**Figure 7:**
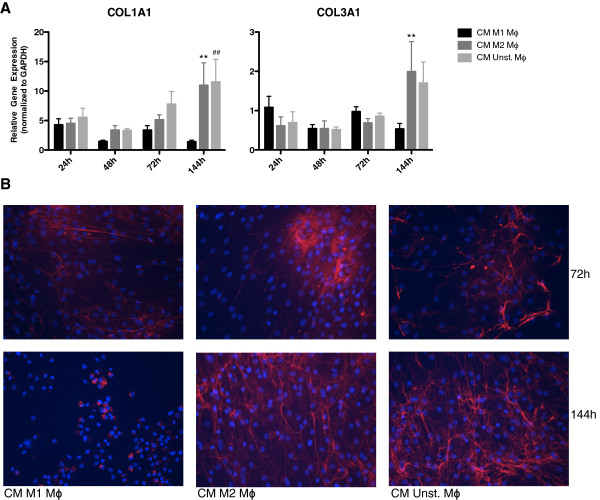
**Influence of CM of M1 polarized, M2 polarized or unstimulated macrophages on extracellular matrix deposition by fibroblasts.** After 144 h, HDFs stimulated with CM of M1 macrophages showed reduced COL1A1 and COL3A1 gene expression levels compared to stimulation with CM of M2 and unstimulated macrophages (**A**). No differences in COL1A1 deposition by fibroblasts were seen after the three different stimulations after 72 h. However, less collagen type I protein deposition was seen in fibroblasts stimulated with CM of M1 macrophages compared to stimulation with CM of unstimulated macrophages after 144 h (**B**). ** p < 0.01, Difference between HDFs stimulated with CM of M1 polarized and CM of M2 polarized macrophages. ## p < 0.01, Difference between HDFs stimulated with CM of M1 polarized and CM of unstimulated macrophages. Gene expression analysis data were analyzed using two-way ANOVA followed by Bonferroni’s post-test, n = 4. COL1A1 protein expression was shown in red, nuclei in blue (DAPI) and original magnification was 200×.

ECM deposition by fibroblasts is an important process in wound healing and fibrosis. Two major collagens produced in these processes are collagen type I (COL1A1) and collagen type III (COL3A1). COL1A1 gene expression in fibroblasts was reduced after stimulation with CM of M1 macrophages compared to CM of M2 and unstimulated macrophages after 144 h (Figure 7A). CM of M1 macrophages reduced COL3A1 gene expression in fibroblasts compared to CM of M2 macrophages at 144 h (Figure 7A). No difference in COL1A1 and COL3A1 gene expression was seen in fibroblasts stimulated with CM of M2 or unstimulated macrophages compared to fibroblasts cultured in control medium (data not shown).

After 72 h, no difference in collagen type I deposition was seen after the different stimulations. However, less collagen type I protein deposition was seen by fibroblasts stimulated with CM of M1 macrophages compared to the other conditions after 144 h (Figure 7B). These results are in accordance with the gene expression patterns of the stimulated fibroblasts.

The results indicate that CM of M1 macrophages reduce ECM deposition by fibroblasts.

### HDFs stimulated with CM of M1 macrophages followed by stimulation with CM of M2 macrophages or non-CM (switch)

In wound healing, the inflammatory phase is normally followed by the healing phase. In both phases macrophages and fibroblasts play an important role. *In vitro*, it is shown that macrophages can be re-polarized from M1 to M2 and *vice versa*[26,27]. *In vivo*, there are indications that re-polarization of macrophages also occurs [28,29]. Therefore we investigated the influence of CM of M1 macrophages on fibroblasts followed by stimulation with CM of M2 macrophages or non-CM at 72 h (24 h CM M1 followed by 48 h CM M2 or non-CM) and 144 h (48 h CM M1 followed by 96 h CM M2 or non-CM).

**Figure 8:**
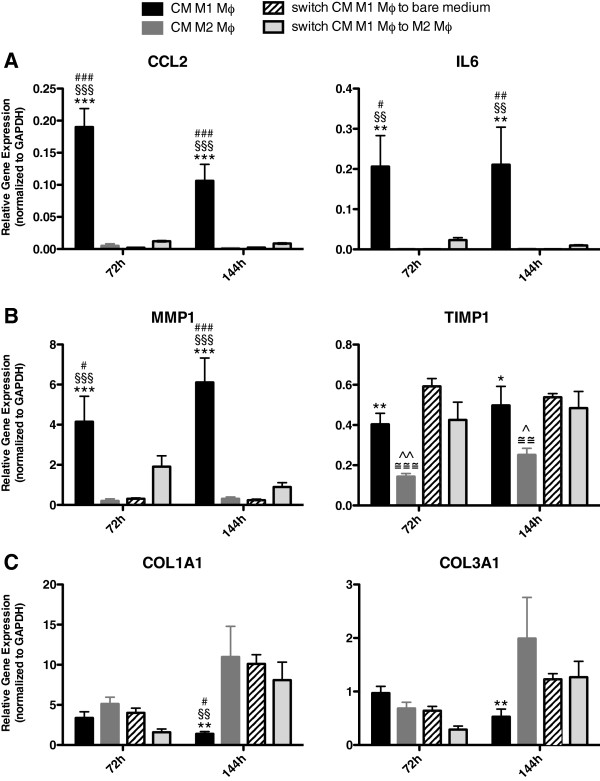
**Fibroblasts stimulated with CM of M1 macrophages followed by stimulation with CM of M2 macrophage or non-CM (switch).** When stimulation of HDFs with CM of M1 macrophages is followed by CM of M2 macrophages or non-CM (switch), the pro-inflammatory genes CCL2 and IL6 were completely downregulated and showed the same gene expression as fibroblasts stimulated with only CM of M2 macrophages after 72 h and 144 h (**A**). MMP1 gene expression after the CM switch was downregulated at 144 h, whereas TIMP1 expression remained similar (**B**). COL1A1 gene expression was upregulated after the CM switch compared to fibroblasts stimulated with CM of M1 macrophages at 144 h. This gene expression level was similar to fibroblasts stimulated with CM of M2 macrophages (**C**). After the switch no differences were seen in COL3A1 gene expression compared fibroblasts stimulated with CM of M1 or CM of M2 macrophages (**C**). * p < 0.05, Difference between HDFs stimulated with CM of M1 polarized and CM of M2 polarized macrophages, ** p < 0.01, *** p < 0.001. §§ p < 0.01, Difference between HDFs stimulated with CM of M1 polarized and the switch to non-CM, §§§ p < 0.001. # p < 0.05, Difference between HDFs stimulated with CM of M1 polarized and the CM switch, ### p < 0.001. ≅≅ p < 0.01, Difference between HDFs stimulated with CM of M2 polarized and the switch to non-CM, ≅≅≅ p < 0.001. ^ p < 0.05, Difference between HDFs stimulated with CM of M2 polarized and the CM switch. Gene expression analysis data were analyzed using two-way ANOVA followed by Bonferroni’s post-test, n = 4.

As shown in Figure 3, fibroblasts became pro-inflammatory after stimulation with CM of M1 macrophages. Figure 8A shows that if this stimulation is followed by CM of M2 macrophages or non-CM, the fibroblasts completely downregulated the gene expression of CCL2 and IL6 both after 72 h and 144 h. The gene expression level of CCL2 and IL6 was similar to fibroblasts stimulated with only CM of M2 macrophages at both time points.

As shown in Figure 4, expression levels of MMP1, MMP2 and MMP14 were upregulated after stimulation of CM from M1 macrophages. Fibroblasts which were stimulated with CM of M1 followed by CM of M2 macrophages or non-CM, showed a downregulation in the gene expression of MMP1 after 72 h and 144 h (Figure 8B). MMP2 expression by fibroblasts after the CM switch showed a slight decrease after 72 h. After 144 h, no differences in MMP2 expression levels were seen between fibroblasts stimulated with CM of M1 or M2 macrophages nor the switch (data not shown). MMP14 gene expression was downregulated in fibroblasts that were stimulated with CM of M1 followed by CM of M2 macrophages or non-CM compared to stimulation with CM of M1 macrophages after 72 h. Similar to the gene expression of MMP2, no differences in MMP14 expression were seen between the conditions after 144 h (data not shown). As shown in Figure 4A, TIMP1 was upregulated in fibroblasts after stimulation with CM of M1 macrophages. Fibroblasts, stimulated with CM of M1 followed by CM of M2 macrophages or non-CM, showed a TIMP1 gene expression that remained high at 72 h and 144 h, which was significantly different compared to fibroblasts stimulated with CM of M2 macrophages alone (Figure 8B), indicating that CM of M2 macrophages nor non-CM was not able to suppress the induction of TIMP expression by CM of M1 macrophages.

ACTA2 gene expression was similar between fibroblasts stimulated with CM of M1 or M2 macrophages or the switch after 72 h. After 144 h fibroblasts stimulated with CM of M2 macrophages or the switch showed higher expression of ACTA2 compared to fibroblasts stimulated with only CM of M1 macrophages (data not shown). No differences were seen in TAGLN gene expression between the three conditions (data not shown).

COL1A1 gene expression was upregulated after the switch of CM compared to fibroblasts stimulated with M1 macrophages CM at 144 h (Figure 8C). This gene expression was similar to fibroblasts stimulated with CM of M2 macrophages after 144 h. No differences in COL3A1 gene expression were seen after the switch compared to fibroblasts stimulated with M1 or M2 CM in time.

The results indicate that the effects of factors produced by M1 macrophages on HDFs diminish once HFDs are not exposed to these factors anymore (i.e. if HDFs are exposed to M2-CM or non-CM).

## Discussion

Macrophages play important roles in wound repair processes. Macrophages are phenotypically highly plastic, and their polarization state depends on the micro-environment present in the wounded area. The M1 and M2 polarization states are opposite activation states of a continuum. Protocols to induce M1 and M2 macrophages *in vitro* are widely used, but it should be realized that the macrophage phenotype in wounds likely exhibit a more complex phenotype in (certain stages of) wound healing [37-39]. Nevertheless, since M1 and M2 macrophages are well-defined extremes, they offer interesting opportunities to study processes encountered during wound healing.

In this study we investigated the influence of secreted factors (conditioned medium) of M1 or M2 macrophages on dermal fibroblasts. Simultaneously, the influence of secreted factors of M1 macrophages followed by stimulation with secreted factors of M2 macrophages was investigated. In addition, we used conditioned medium from unstimulated macrophages. These unstimulated macrophages have a “M2-like” phenotype, which is probably caused by stimulating monocytes with macrophage colony-stimulating factor (M-CSF), a step that is necessary to induce differentiation of monocytes towards macrophages [40,41]. Despite this, the obtained macrophages changed their polarization status quickly when stimulated with LPS/IFNG or IL4/IL13 towards M1 or M2 macrophages, respectively. Secreted factors of these three types of macrophages influenced fibroblasts morphology and phenotype considerably.

In general, macrophages that invade the tissue in the inflammatory phase of wound healing adopt a M1 phenotype. In our model, the secreted factors from M1 macrophages influences the properties of dermal fibroblasts already within 24 h, changing the phenotype into a pro-inflammatory state. This indicates that fibroblasts, under the direction of paracrine signals of M1 macrophages, contribute to a pro-inflammatory environment by secreting cytokines and chemokines (such as CCL2, CCL7 and IL6) in the inflammatory phase of wound healing. This is in accordance with data shown by Holt *et al.*[34]. These authors showed, in an *in vitro* model with murine primary cells and cell lines, that fibroblasts produce pro-inflammatory cytokines and chemokines after stimulation with conditioned medium of LPS-stimulated macrophages and in a co-culture system with direct cell-cell contact. Other studies [30,32,33] showed that after direct contact between macrophages and fibroblasts, without paying attention to the M1/M2 status of macrophages, fibroblasts upregulated the inflammatory proteins CCL2 and CCL3, which is in accordance to our results from fibroblasts stimulated with secreted factors from M1 macrophages.

MMPs are capable of regulating chemokine activity and ECM degradation in tissue repair [42,43]. MMPs are important as they support cellular influxes, but an excess of MMPs will damage the tissue architecture and a high TIMP/MMP ratio is often seen in non-healing tissues. In the inflammation phase of tissue repair MMPs are upregulated and the moment fibroblasts deposit new ECM the MMPs levels decline. In our model we showed that different MMPs (MMP1, MMP2, MMP3 and MMP14) were highly upregulated in fibroblasts that were exposed to paracrine factors derived from M1 macrophages. Because of the secreted MMPs and the pro-inflammatory state of fibroblasts after M1 stimulation, it is likely that *in vivo* the fibroblasts are able to prolong the inflammation state in wound healing by itself or by attracting more pro-inflammatory cells.

Fibroblasts exposed to conditioned medium from M2 macrophages showed little response. Only a slight increase was seen in the expression of ACTA2, but this did not resulted in myofibroblast formation. Furthermore, an increase in cell proliferation was seen, which was in accordance with previous findings [22,23,31,44].

In wound repair it is thought that M2 macrophages are responsible for reversing the inflammatory response, thereby initiating the healing process. Interestingly, in this study we show that fibroblasts with an inflammatory phenotype (initiated by stimulation with secreted factors of M1 macrophages) can be reversed to an anti-inflammatory phenotype with secreted factors of M2 macrophages or non-CM. In these fibroblasts, the previously upregulated pro-inflammatory cytokines, chemokines, and MMPs were completely downregulated after stimulation with paracrine signals from M2 macrophages or non-CM. Thus, although paracrine factors of M2 macrophages have relatively little effect on unstimulated fibroblasts, they can have a major effect on fibroblasts with an inflammatory phenotype.

## Conclusions

In summary, we have shown that secreted factors from M1 macrophages gives rise to fibroblasts with a pro-inflammatory and ECM-degrading profile, while M2 macrophages induce fibroblast proliferation. The pro-inflammatory and ECM-degrading fibroblast can be reversed completely by secreted factors from M2 macrophages or non-CM. Therefore, not only macrophages, but also fibroblasts show a high dynamic plasticity in wound healing / tissue repair processes, a plasticity that seems to be regulated by the micro-environment.

## Material and methods

### Isolation of CD14^+^ cells

Human peripheral blood mononuclear cells (PBMCs) from healthy donors were isolated from buffy coats (Sanquin, Groningen, the Netherlands) by density-gradient centrifugation using Lymphoprep (Axis-Shield, Oslo, Norway) according to the manufacturer’s protocol. Briefly, blood was diluted three times with isolation buffer (pH 7.4) consisting of phosphate buffered saline (PBS) with 0.5% fetal bovine serum (FBS; Life Technologies Europe BV, Bleiswijk, the Netherlands) and 2 mM EDTA (Merck, Darmstadt, Germany). This mixture (30 ml) was layered over 20 ml of Lymphoprep and centrifuged at 800 × g for 30 min. Residual erythrocytes were lysed on ice (10 min) in 155 mM NH_4_Cl, 10 mM KHCO_3_, 0.1 mM EDTA (pH 7.4) and the suspension was centrifuged at 300 × g at 4°C for 10 min after which the supernatant was discarded and the pellet gently resuspended in isolation buffer. PBMCs were counted using a Coulter Counter (Beckman Coulter, Inc. Brea, USA).

CD14^+^ cells were isolated by immunomagnetic bead separation using CD14 Microbeads (Miltenyi Biotec B.V., Leiden, the Netherlands). Briefly, 1 × 10^7^ PBMCs were labeled with 20 μl CD14 Microbeads and incubated on ice in 80 μl isolation buffer for 30 min. Cells were washed with isolation buffer and the suspension was centrifuged at 300 × g at 4°C for 10 min. The pellet was resuspended in degassed isolation buffer and the CD14^+^ cells were separated with an LS column (Miltenyi Biotec B.V., Leiden, the Netherlands) placed on a column adapter in a strong magnetic field. CD14^+^ cells bind to the column and after carefully washing with degassed isolation buffer and removal of the LS column from the magnet the CD14^+^ cells were flushed out from the column using a plunger. The CD14^+^ cells were counted with a Coulter Counter and after centrifugation at 300 × g for 10 min at 4°C gently resuspended in culture medium, consisting of X-VIVO-10 medium (Lonza, Basel, Switzerland) supplemented with 2 mM l-glutamine (Sigma-Aldrich, St. Louis, USA), 1% penicillin/streptomycin (Sigma-Aldrich, St. Louis, USA) and 10 ng/ml recombinant human M-CSF (R&D Systems, Minneapolis, USA).

### Macrophage cell culture, polarization with M1 or M2 stimuli and collection of conditioned media

Immediately after isolation and counting, the cell suspension was plated with a density of 100,000 cells/ cm^2^ onto tissue culture polystyrene plates (TCPS; Corning Incorporated, NY, USA). Cells were cultured at 37°C under 5% CO_2_. Cells were refed at day 3 and non-attached cells were removed from culture at day 6.

At day 6, the adherent cells (macrophages) were washed and stimulated in culture medium (but without M-CSF), with either (1) 1 μg/ml LPS (Sigma-Aldrich, St. Louis, USA) + 10 ng/ml IFNG (PeproTech, Rocky Hill, USA) (classical stimuli), (2) 2 ng/ml IL4 + 2 ng/ml IL13 (both R&D, Minneapolis, USA) (alternative stimuli), or (3) no stimulation (control) at 37°C for 48 h. The polarization state of the macrophages was determined by quantitative RT-PCR (qRT-PCR). The cells were subsequently washed and cultured in X-VIVO-10 medium for 4 h (conditioned medium). After 4 h the CM from (1) M1 macrophages, (2) M2 macrophages and (3) unstimulated macrophages was collected and stored for further analyses at −20°C. The CM of the different conditions were used for stimulation of HDFs, the determination of CCL2 and CCL18 levels by means of enzyme-linked immunosorbent assays (ELISA) and the determination of cytokines with a multiplex bead immunoassay.

### HDF cell culture and stimulation with CM of M1, M2 and unstimulated macrophages

Primary HDFs (#2320, ScienCell, Carlsbad, USA) were seeded onto TCPS overnight with a density of 15,000 cells/cm^2^ in X-VIVO-10 medium containing 2 mM l-glutamine, 1% penicillin/streptomycin and 50 μg/ml l-ascorbic acid 2-phosphate sesquimagnesium salt hydrate (Sigma-Aldrich, St. Louis, USA). The next day the X-VIVO medium was replaced by CM derived of M1, M2 or unstimulated macrophages, which was supplemented with l-ascorbic acid 2-phosphate sesquimagnesium salt hydrate. Passage 5 or 6 of HDFs were used for stimulations with CM from macrophages. The CM was refreshed every day and the stimulated HDFs were characterized at 24 h, 48 h, 72 h and 144 h by morphology, qRT-PCR and after 24 h, 72 h and 144 h by immunofluorescent stainings. The deposition of the extracellular matrix protein collagen type I was determined at 72 h and 144 h. After 24 h and 48 h, CM of stimulated HDFs was collected and stored for further analysis at −20°C. Prior to collection of the CM, the stimulated HDFs were washed and cultured in X-VIVO-10 medium for 4 h. CCL2, CCL7, IL6, MMP1, MMP2 and MMP3 secretion by HDFs was determined by ELISA. All culture conditions were carried out at 37°C under 5% CO_2_.

### Stimulation of HDFs by CM of M1 macrophages followed by stimulation with CM of M2 macrophages (switch)

HDFs were cultured as described above. After overnight seeding in X-VIVO-10 medium the medium was replaced by CM of M1 macrophages for 24 h or 48 h, with refreshment of the CM after 24 h. After 24 h or 48 h the medium was replaced by CM of M2 macrophages or by X-VIVO-10 medium (non-CM) for another 48 h or 96 h, respectively (total culture time now 72 h and 144 h, respectively); the CM or non-CM were refreshed every day. The HDFs were characterized by qRT-PCR.

### RNA isolation, cDNA synthesis and qRT-PCR

Total RNA was isolated from the cells using the RNeasy Kit (Qiagen Inc., CA, USA) in accordance to the manufacturer’s protocol. RNA concentration and purity were determined by UV spectrophotometry (NanoDrop Technologies, Wilmington, NC). For qRT-PCR analysis, total RNA was reverse transcribed using the First Strand cDNA synthesis kit (Fermentas UAB, Lithuania) in accordance to the manufacturer’s protocol. Quantification of gene expression was performed using qRT-PCR analysis in a final reaction volume of 10 μl, consisting of 1× SYBR Green Supermix (Bio-Rad, Hercules, USA), 6 μM forward primer, 6 μM reverse primer (Table 2) and 5 ng cDNA. Reactions were performed at 95°C for 15 sec, 60°C for 30 sec, 72°C for 30 sec, for 40 cycles in a ViiA™ 7 Real-Time PCR System (Applied Biosystems, CA, USA). Analysis of the data was performed using ViiA 7™ Real-Time PCR System Software v1.1 (Applied Biosystems, CA, USA).

**Table 2:** Overview of primers used for qRT-PCR analysis

**Target gene**	**Forward sequence**	**Reverse sequence**
ACTA2	CTGTTCCAGCCATCCTTCAT	TCATGATGCTGTTGTAGGTGGT
CCL18	ATGGCCCTCTGCTCCTGT	AATCTGCCAGGAGGTATAGACG
CCL2	AGTCTCTGCCGCCCTTCT	GTGACTGGGGCATTGATTG
CCL7	ATGAAAGCCTCTGCAGCACT	TCTGTAGCAGCAGGTAGTTGAAGT
CD14	AGCTAAAGCACTTCCAGAGC	AGTTGTGGCTGAGGTCTAGG
CD40	GGTCTCACCTCGCTATGGTT	CAGTGGGTGGTTCTGGATG
CD68	GTCCACCTCGACCTGCTCT	CACTGGGGCAGGAGAAACT
CLEC10A	AGGGTTTCAAGCAGGAACG	AGGTGTGCCTTCTGCGTAGT
COL1A1	GCCTCAAGGTATTGCTGGAC	ACCTTGTTTGCCAGGTTCAC
COL3A1	CTGGACCCCAGGGTCTTC	CATCTGATCCAGGGTTTCCA
GAPDH	AGCCACATCGCTCAGACAC	GCCCAATACGACCAAATCC
IL1B	TACCTGTCCTGCGTGTTGAA	TCTTTGGGTAATTTTTGGGATCT
IL1R2	TTTCTGCCTTCACCCTTCAG	GGCACCTCAGGGCTACAG
IL6	ACTTGCCTGGTGAAAATCAT	CAGGAACTGGATCAGGACTT
MMP1	GCTAACCTTTGATGCTATAACTACGA	TTTGTGCGCATGTAGAATCTG
MMP14	TACTTCCCAGGCCCCAAC	GCCACCAGGAAGATGTCATT
MMP2	CCCCAAAACGGACAAAGAG	CTTCAGCACAAACAGGTTGC
MMP3	CAAAACATATTTCTTTGTAGAGGACAA	TTCAGCTATTTGCTTGGGAAA
MRC1	ACACCAAAACCTGAGCCAAC	CCACCCATCTTCAGTAACTGGT
TAGLN	CTGTTCCAGCCATCCTTCAT	TCATGATGCTGTTGTAGGTGGT
TIMP1	GAAGAGCCTGAACCACAGGT	CGGGGAGGAGATGTAGCAC
YWHAZ	GATCCCCAATGCTTCACAAG	TGCTTGTTGTGACTGATCGAC

### Enzyme-linked immunosorbent assay (ELISA)

Determination of CCL2, CCL7, CCL18, IL6, MMP1, MMP2 and MMP3 protein levels were measured using DuoSet® ELISA Development kit (R&D Systems, Minneapolis, USA) in accordance to manufacturer’s protocol. Briefly, 96 wells plates (#9018, Corning, Amsterdam, The Netherlands) were coated with Capture Antibody and incubated overnight at room temperature (RT). After incubation the plates were washed with 0.05% Tween-20 (Sigma-Aldrich, St. Louis, USA) in PBS and blocked with 1% bovine serum albumin (BSA) (Sanquin, Amsterdam, the Netherlands) in PBS for 1 h. After washing, the plates were incubated with diluted sample or matched standards for 2 h. The detection was performed using matched biotin conjugated antibodies followed by streptavidin-poly-horseradish peroxidase (Sanquin, Amsterdam, The Netherlands). The color reaction was performed with tetramethylbenzidine (TMB; Sigma-Aldrich, St. Louis, USA) in sodium acetate buffer, pH 6, containing H_2_O_2_ and stopped with 1 M H_2_SO_4_. The absorbance was measured using a microplate reader (VERSA max, Molecular Devices Inc., CA, USA). The detection limit for MMP2, MMP1, MMP3, CCL2, IL6, CCL7 and CCL18 was 312 pg/ml, 78 pg/ml, 15.6 pg/ml, 7.8 pg/ml, 4.7 pg/ml and 3.9 pg/ml, respectively.

### Multiplex bead immunoassay

Factors that were secreted by M1, M2 and unstimulated macrophages were determined by a multiplex bead immunoassay in accordance to manufacturer’s protocol (Invitrogen Corporation, Carlsbad, USA). Briefly, beads that have defined spectral properties and are conjugated to protein-specific capture antibodies were added to a 96 well filter plate. After washing, the plate was incubated with sample or matched standards for 2 h. The detection was performed using protein-specific biotinylated detector antibodies and streptavidin conjugated R-Phycoerythrin. The beads were analyzed with the Luminex-100 detection system (Luminex, Austin, USA).

### Proteolytic activity assay

MMP activity was determined in the CM of HDFs after 24 h of stimulation with CM derived of M1, M2 or unstimulated macrophages. The CM of the HDFs was mixed, in a black 96 flat bottom plate, with prewarmed assay buffer containing 0.1 M 4-(2-hydroxyethyl)-1-piperazineethanesulfonic acid (HEPES), 20 mM CaCl_2_, 0,1% Brij-35, pH 7.0 and 10 μM OmniMMP™ fluorogenic substrate (BML-P126, Enzo Life Sciences, Antwerpen, Belgium). The fluorescent intensity was measured using a fluorescence plate reader (BIO-TEK FL600, BIO-TEK instruments, Inc., Winooski, USA) after 20 h of incubation at 37°C.

### Immunofluorescent stainings for ACTA2 and MKI67 on stimulated adult human dermal fibroblasts

After 24 h and 144 h of culture, HDFs were washed twice with PBS and fixed in 2% paraformaldehyde (PFA) at RT for 10 min. Fixed cells were incubated with 0.5% Triton X-100 (Merck, Darmstadt, Germany) in PBS for 3 min at RT. After washing with PBS the cells were incubated with (1) mouse-anti-human ACTA2 (M0851, Dako, Glosstrup, Denmark) (1:100) or (2) rabbit-anti-human MKI67 (MONOSAN®, Uden, The Netherlands) (1:500) diluted in PBS containing 1% BSA for 1 h at RT. After three washes with PBS, cells were incubated with biotinylated (1) goat-anti-mouse IgG2a-biotin (SouthernBiotech, Alabama, USA) (1:100), or (2) goat-anti-rabbit-FITC (SouthernBiotech, Alabama, USA) (1:100) diluted in PBS containing 2% normal human serum (NHS) for 30 min at room temperature. The cells were subsequently washed three times with PBS and incubated with streptavidine-CY3 (Invitrogen, Grand Island, USA) (1:100) in PBS containing 1% BSA, 2% NHS and DAPI (1:5000) for 30 min. After three washes with PBS the slides were mounted in Citifluor (Agar Scientific, Essex, UK) and examined by immunofluorescent microscopy using a Leica DMRA microscope equipped with a Leica DFC350FX digital camera and Leica Application Suite (LAS) software (all Leica Microsystems, Wetzlar, Germany).

### Collagen type I deposition by HDFs after stimulation with CM of M1, M2 or unstimulated macrophages

After 72 h and 144 h of culture, HDFs were washed twice with PBS and fixed in 2% PFA at RT for 10 min. Fixed cells were incubated at RT with (1) mouse-anti-human collagen type I (COL I) (1:100) (ab90395, Abcam, Cambridge, UK) diluted in PBS containing 1% BSA for 1h. The HDFs were washed three times with PBS, followed by incubation with goat-anti-mouse IgG1-biotin (SouthernBiotech, Alabama, USA) (1:100) diluted in 1% BSA in PBS for 30 min. The cells were subsequently washed three times with PBS and incubated with streptavidine-CY3 (1:100) in PBS containing 1% BSA, 2% NHS and DAPI (1:5000) for 30 min. After three washes with PBS the slides were mounted in Citifluor and examined by immunofluorescent microscopy using a Leica DMRA microscope.

### Collagen gel contraction

Collagen gels were prepared by mixing X-VIVO-10 medium, 1 M NaOH, 10 × PBS, 0.2 M HEPES and collagen I (BD Biosciences, Franklin Lakes, NJ USA). The final concentration was 5.2 mM NaOH, 1 × PBS, 2 mM HEPES, 2.4 mg/ml of collagen I in X-VIVO-10 medium. HDFs were added in a concentration of 200.000 cells/ml and 500 μl of this mixture was pipetted into a well of a 24-well culture plate. Polymerization of the solution occurred within 1h at 37°C under 5% CO_2_. After polymerization CM of M1, M2 or unstimulated macrophages was added. As control complete X-VIVO medium supplemented with 10 ng/ml TGFB1 (PeproTech EC Ltd, London, UK) was used. The CM and medium supplemented with TGFB1 was refreshed every day and the cells were cultured at 37°C under 5% CO_2_. After 5 days the gels were gently released and contractile force was analyzed by measuring the gel diameter at 8 h after release using a flatbed scanner (Hewlett-Packard Company, Palo Alto, USA) Data are expressed as the percentage of area compared to the initial gel area.

### Statistics

All data are represented as means ± standard error of the mean of at least three independent experiments and were analyzed by Graph-Pad Prism Version 5 for Macintosh (GraphPad Software, Inc., La Jolla, CA, USA) either by one-way ANOVA followed by Tukey’s post hoc analysis, or by two-way ANOVA followed by Bonferroni post hoc analysis. Values of *P* < 0.05 were considered to be statistically significant.

## Abbreviations

ACTA2: Alpha-actin-2; BSA: Bovine serum albumin; CCL: Chemokine (C-C motif) ligand; CLEC10A: C-type lectin domain family 10, member A; CM: Conditioned medium; COL1A1: Collagen type I; COL3A1: Collagen type III; CXCL: Chemokine (C-X-C motif) ligand; ECM: Extracellular matrix; FBS: Fetal bovine serum; FGF2: Fibroblast growth factor 2; HDFs: Human primary dermal fibroblasts; IFNG: Interferon gamma; IL: Interleukin; LPS: Lipopolysaccharides; M-CSF: Macrophage colony-stimulating factor; M1: Classically activated macrophages; M2: Alternatively activated macrophages; MKI67: Nuclear protein Ki-67; MMP: Matrix metalloproteinase; MRC1: Mannose receptor, C type 1; NHS: Normal human serum; PBMCs: Peripheral blood mononuclear cells; PBS: Phosphate buffered saline; PFA: Paraformaldehyde; qRT-PCR: *Q*uantitative *R*everse *T*ranscription polymerase chain reaction; TAGLN: Transgelin; TCPS: Tissue culture polystyrene plates; TGFB1: Transforming growth factor beta 1; TIMP1: Tissue inhibitor of metalloproteinases −1; TLR4: Toll-like receptor 4; TMB: Tetramethylbenzidine; TNF: Tumor necrosis factor; YWHAZ: Tyrosine 3-monooxygenase/tryptophan 5-monooxygenase activation protein, zeta polypeptide.

## Competing interests

The authors declare that they have no competing interests.

## Authors’ contributions

DTAP designed research, performed experiments, analyzed data, interpreted data and wrote the manuscript. NAH designed research, performed experiments, interpreted data and edited the manuscript. MS contributed to preliminary experiments. JAK and SdR performed experiments and edited the manuscript. R.A.B. supervised the work, interpreted the data, and edited the manuscript. All authors read and approved the final manuscript.
